# Crosstalk between autophagy and metabolism: implications for cell survival in acute myeloid leukemia

**DOI:** 10.1038/s41420-024-01823-9

**Published:** 2024-01-24

**Authors:** Yongfeng Chen, Jia Chen, Zhenyou Zou, Linglong Xu, Jing Li

**Affiliations:** 1 Department of Basic Medical Sciences, Medical College of Taizhou University, 318000 Taizhou, Zhejiang China; 2 School of Medicine, Zhejiang University, 310058 Hangzhou, Zhejiang China; 3 Brain Hospital of Guangxi Zhuang Autonomous Region, 542005 Liuzhou, Guangxi China; 4 Department of Hematology, Taizhou Central Hospital (Taizhou University Hospital), 318000 Taizhou, Zhejiang China; 5 Department of Histology and Embryology, North Sichuan Medical College, 637000 Nanchong, Sichuan China

**Keywords:** Macroautophagy, Acute myeloid leukaemia

## Abstract

Acute myeloid leukemia (AML), a prevalent form of leukemia in adults, is often characterized by low response rates to chemotherapy, high recurrence rates, and unfavorable prognosis. A critical barrier in managing refractory or recurrent AML is the resistance to chemotherapy. Increasing evidence indicates that tumor cell metabolism plays a crucial role in AML progression, survival, metastasis, and treatment resistance. Autophagy, an essential regulator of cellular energy metabolism, is increasingly recognized for its role in the metabolic reprogramming of AML. Autophagy sustains leukemia cells during chemotherapy by not only providing energy but also facilitating rapid proliferation through the supply of essential components such as amino acids and nucleotides. Conversely, the metabolic state of AML cells can influence the activity of autophagy. Their mutual coordination helps maintain intrinsic cellular homeostasis, which is a significant contributor to chemotherapy resistance in leukemia cells. This review explores the recent advancements in understanding the interaction between autophagy and metabolism in AML cells, emphasizing their roles in cell survival and drug resistance. A comprehensive understanding of the interplay between autophagy and leukemia cell metabolism can shed light on leukemia cell survival strategies, particularly under adverse conditions such as chemotherapy. This insight may also pave the way for innovative targeted treatment strategies.

## Facts

- Autophagy plays a crucial role in maintaining cellular metabolic balance during nutritional supply, growth factor, and energy changes.
- The crosstalk between autophagy and metabolism plays a key role in maintaining cellular homeostasis in tumor cells.
- Bifocal interventions targeting tumor energy metabolism and autophagy have emerged as a new therapeutic strategy.

## Open questions

- How to definitively determine whether autophagy plays a protective or harmful role in different types and stages of AML?
- How can we exploit the process of autophagy to develop effective therapeutic strategies for AML?

## Introduction

Acute myeloid leukemia (AML) is a biologically intricate and profoundly challenging disease, characterized by its ability to adapt to adverse conditions, thereby enhancing survival and growth [1–3]. This adaptability is intrinsically linked to the energy metabolism of AML cells, which not only demand energy but also biosynthetic precursors for relentless proliferation [4]. In recent years, the phenomenon of metabolic reprogramming in AML cells, as a survival and drug resistance mechanism, has received considerable attention. In this context, autophagy, a cellular process that enables cells to remove damaged organelles timely and provide energy for normal functions, emerges as a critical player [5].

Although the precise mechanisms underlying the regulation of metabolic reprogramming in AML cells by autophagy remain somewhat unclear, it is evident that autophagy plays a crucial role in this metabolic adaptation. Contemporary research has demonstrated that autophagy is not only vital in maintaining cellular homeostasis but also in the occurrence, development, treatment, and prognosis of cancer [6, 7]. In leukemia research, the influence of autophagy on leukemia cell proliferation, invasion, metastasis, and apoptosis has been extensively studied, revealing significant findings. The spotlight is now on elucidating the role of autophagy in the regulation of energy metabolism in leukemia cells, particularly glucose, lipid, and amino acid metabolism [8, 9].

In this review, we aim to present an overview of the most recent research advancements in understanding the role of autophagy in the metabolic reprogramming of AML cells. We delve into the impact and mechanisms of autophagy on the regulation of these metabolic pathways, highlighting potential therapeutic strategies that target the interplay between autophagy and metabolic reprogramming in AML.

## Autophagy and hematopoiesis

Autophagy is a self-degradative process that is essential for balancing sources of energy at critical times in development and in response to nutrient stress. It is a lysosome-dependent intracellular degradation pathway that allows for the processing and recycling of large biomolecules and damaged organelles. There are several types of autophagy, each with distinct mechanisms and roles: chaperone-mediated autophagy (CMA), microautophagy, and macroautophagy [10]. CMA involves the direct translocation of substrates across the lysosomal membrane facilitated by chaperone proteins. Microautophagy, on the other hand, entails the direct invagination of the lysosomal membrane to sequester cytoplasmic content. The most extensively studied form, macroautophagy, involves the severing of portions of the cytoplasm within double-membraned vesicles known as autophagosomes, which subsequently fuse with lysosomes to form autolysosomes where the engulfed material is degraded and recycled. Upon the fusion of autophagosomes with lysosomes, the inner autophagosome membrane is rapidly degraded, leading to the release of the enclosed substrates that are efficiently hydrolyzed by a host of lysosomal enzymes. Through this complex process, a diverse array of catabolic products, including amino acids, nucleotides, and fatty acids, are liberated into the cytosol from autolysosomes. These resultant molecules can then be repurposed and funneled into various bioenergetic and biosynthetic pathways, which is especially crucial during states of cell stress or nutrient paucity [11, 12].

Mounting evidence has suggested that in normal hematopoiesis, high autophagic activity is critical for maintaining hematopoietic stem and progenitor function. According to Gomez-Puerto et al., the knockdown of autophagy genes ATG5 or ATG7 leads to decreased hematopoietic stem and progenitor cell (HSPC) frequencies both in vitro and in vivo. This may be due to reduced cell cycle progression and increased apoptosis, which are associated with increased expression of proapoptotic genes such as BCL-2-associated X, apoptosis regulator (BAX), and p53 upregulated modulator of apoptosis (PUMA) and the cell cycle inhibitor, p21. Additionally, there are increased levels of reactive oxygen species (ROS) [13]. In mice, deficiency in the Atg5 gene in hematopoietic cells impairs autophagy-mediated clearance of damaged mitochondria. This results in an increase in ROS levels, which in turn leads to functional defects in hematopoietic stem cells (HSCs) and causes a disruption in the differentiation of mature progenitor and terminally differentiated cells. Ultimately, this causes a significant decrease in the absolute numbers of hematopoietic stem cells and multipotent progenitor cells [14]. Furthermore, numerous studies have also confirmed that autophagy is an important regulatory mechanism for HSCs. It plays a crucial role in preventing the accumulation of damaged mitochondria, protecting HSCs from DNA oxidative damage, maintaining self-renewal and differentiation of HSCs, and inhibiting cellular malignant transformation [15–17].

In addition to maintaining normal hematopoiesis, autophagy may also play a role in the initiation and development of AML, according to several lines of evidence. In Atg7-deficient mice, the loss of autophagy in hematopoietic stem cells not only leads to increased aberrant myeloid expansion, resulting in severe myeloid dysplasia and infiltrating myeloid blast cells evoking AML [18] but can also activate the NOTCH signaling pathway, blocking HSC differentiation and leading to a leukemic phenotype [19]. These findings suggest that autophagy is an important anticancer mechanism that can restrict the transformation of normal hematopoietic cells into leukemia cells. A widely accepted view is that autophagy serves as a quality control mechanism during the early stages of leukemia development, acting to suppress leukemia initiation. Should autophagy become impaired, it may drive the initiation and development of leukemia [20].

Nevertheless, upon transitioning into the leukemic state, autophagy may serve as a protective mechanism for leukemia cells. Autophagy degrades and recycles damaged cellular components and plays a key role in the metabolic reprogramming of cells, helping them cope with adverse environmental stimuli [21]. Moreover, by clearing damaged mitochondria, autophagy averts excessive build-up of ROS in cells, thereby shielding leukemia cells from oxidative stress [22]. According to a study by Sumitomo et al., autophagy-deficient leukemia-initiating cells (LICs) in Atg5 or Atg7-deficient mouse models of AML exhibited enhanced mitochondrial activity and increased ROS production. This was accompanied by an increase in cell death, suggesting that the survival of LICs is critically dependent on autophagy [23]. Notably, strategies to modulate autophagy—either through stimulation or suppression—have been widely tested in innovative treatments for AML, showing promising results and potentially paving the way toward enhanced clinical outcomes [3, 24–32] (Table 1).

**Table 1:** Emerging autophagy modulation in AML treatment.

Methods	Drug used	Anti-AML effect in pre-clinic research	Ref.
Autophagy inhibition	3-methyladenine	Increase the sensitivity of AML cells to cytarabine both in vitro and in vivo	[24]
	Chloroquine	Enhances the toxicity of AraC to AML cells but appears to be ineffective for patients who have relapsed and for AraC-resistant AML cells	[3]
	Bafilomycin	Potentiates the cytotoxic effects of ATO/ATRA in AML cell lines KG-1 and HL-60	[25]
	Wortmannin	Impairs expression of anti-apoptotic Bcl-2 family members Bcl-2 and BclXL in primary AML cells via blockade of PI3K/AKT signaling	[26]
Autophagy induction	Metformin	Inhibits Acute Myeloid Leukemia Cell Growth through the AMPK/mTOR Pathway and Autophagic Regulation	[27]
	Resveratrol	Modulates autophagy and induces apoptosis in HL-60 cells	[28]
	Quercetin	Induces autophagy-associated death in HL-60 cells through CaMKKβ/AMPK/mTOR signal pathway	[29]
	Neratinib	Suppresses proliferation and promotes apoptosis of HL-60 cells through autophagy-dependent ferroptosis	[30]
	ATRA	Promotes differentiation in APL cells via autophagy induction	[31]
	ATPR	Triggers ferroptosis and promotes AML differentiation through the regulation of autophagy via iron homeostasis	[32]

*APL* acute promyelocytic leukemia, *ATO* arsenic trioxide, *ATPR* 4-Amino-2-Trifluoromethyl-Phenyl Retinate, *ATRA* all-trans retinoic acid, *AraC* cytarabine.

## Interplay between autophagy and glycolysis in AML cells

Warburg and his colleagues uncovered that various tumors produce lactate regardless of the presence of oxygen. This led to the proposal of the Warburg effect, which signifies that tumor metabolism employs aerobic glycolysis even under oxygen-sufficient conditions [33]. While glycolysis is less efficient than oxidative phosphorylation at deriving ATP from glucose, it offers several benefits to the energy metabolism of tumor cells. It not only rapidly produces ATP, but also provides abundant substrates for cellular anabolic metabolism, reduces ROS generation through the pentose phosphate pathway, and produces excessive lactate that acidifies the extracellular environment, thereby promoting the survival and invasion of cancer cells [34, 35].

Multiple studies have demonstrated that the dependence of AML on increased glycolysis is no different from other malignancies [36–38]. Accordingly, patients with AML were found to display an abnormal glucose metabolism signature, and several glucose metabolite biomarkers had been implicated as prognostic values [36]. Previous research has shown that glycolysis, as the main energy source for tumors, has a significant impact on the initiation of AML, as well as the survival, proliferation, invasiveness, and drug resistance of AML cells [39, 40]. Recently, Chen et al. reported that AML cells exhibited higher levels of glycolysis under glucose-rich conditions and key glycolysis-related genes, glucose transporter 1 (GLUT1) and monocarboxylate transporter 1 (MCT1), were upregulated. The abnormally increased glucose effectively promoted leukemia cell proliferation and inhibited apoptosis. Intervention with 2-deoxy-D-glucose (2-DG) reversed the glycolysis induced by glucose treatment, and pharmacological blockade of glucose uptake improved the condition of leukemia [40]. The rapid proliferation of AML cells can gradually lead to the depletion of glucose in the bone marrow. However, AML cells can upregulate the expression of the Glucose Transporter 5 (GLUT5) protein, allowing them to use fructose as an alternative substrate for glycolysis. Pharmacological blockade of fructose uptake enhances the condition of leukemia and augments the cytotoxicity of the leukemia drug Cytarabine (Ara-C) [41]. Furthermore, studies have shown that deletion of glycolysis enzymes pyruvate kinase isoform M2 (PKM2) and lactate dehydrogenase A (LDHA) delays leukemia progression [39], and the inhibition of glycolysis can significantly suppress AML cell proliferation and promote apoptosis [36, 37]. Besides, multiple studies also highlight that inhibition of key glycolysis enzymes would be a vital adjuvant therapy strategy for AML [38, 42–48] (Table 2).

**Table 2:** Targeting key glycolysis enzymes in AML.

Drug target	Drug used	Anti-AML effect in pre-clinic research	Ref.
HK	2-DG	Reducing metabolic activity, inducing apoptosis and cell cycle arrest in various AML cell lines	[38]
	3-BrP	Exhibiting synergistic inhibitory effects with DNR on THP-1 AML cell lines *via* S-phase and G2/M-phase cell cycle arrest	[42]
	Lonidamine	Promoting the anti-AML effect of matrine both in vitro and in vivo	[43]
PFK2	PFK15	Synergistically inhibiting the proliferation of mTOR hyper-activated AML cells with rapamycin	[44]
PKM2	Shikonin	Induces cytotoxicity in AML cells *via* ROS-dependent mechanism and spares normal cells. Impairs oxidative TCA cycling and reprograms AML cells towards glycolysis	[45]
	MCL	Exerts selective cytotoxic effects on AML LSC via inhibition of NF-κB expression and activity, and by generating intracellular ROS	[46]
PDK1	DAP	Inhibits cell proliferation, induces apoptosis, and suppresses autophagy in AML cells by modulating multiple signaling pathways, particularly the PI3K/Akt pathway	[47]
G6PD	6AN	Effectively suppresses growth, migration, and invasion of AML by increasing fatty acid oxidative oxidation and decreasing glucose oxidation	[48]

*2-DG* 2-deoxy-D-glucose, *3-BrP* 3-Bromopyruvate, *6AN* 6-aminonicotinamide, *DAP* 2,2-dichloroacetophenone, *DNR* daunorubicin, *G6PD* glucose-6-phosphate dehydrogenase, *HK* hexokinase, *MCL* micheliolide, *PDK* pyruvate dehydrogenase kinase, *PFK2* phosphofructo-kinase 2, *PKM2* pyruvate kinase isoform M2, TCA, tricarboxylic acid.

Several studies indicate that tumor cell metabolic reprogramming is governed by both inherent genetic alterations, such as the loss of tumor suppressors or the activation of oncogenes, and external influences from the tumor microenvironment, such as hypoxia, ROS, and other factors [49–52]. However, studies have indicated that a high glycolysis flux also correlates with a decreased level of autophagy, leading to more aggressive leukemias in vivo. Using human AML bone marrow mononuclear cells (BM-MNCs) and an AML mouse model, Watson et al. found that the glycolysis and proliferation of AML cells were inhibited by autophagy. A heterozygous loss of Atg5 in AML cells results in a decrease in autophagy flux, leading to a glycolytic shift and promoting the proliferation of AML cells both in vitro and in vivo [53]. Similarly, Baker et al. reported that shRNA-mediated ATG3 deletion impaired the autophagy function of AML cells, leading to the upregulation of glycolysis, lactate production, and mitochondrial metabolism in AML cells. Furthermore, the deletion of ATG3 sensitized AML cells to the inhibition of oxidative phosphorylation (OXPHOS), highlighting the metabolic vulnerabilities that leukemia cells acquire from autophagy inhibition [54].

The regulation of glycolysis by autophagy is not yet fully understood. PKM2 is a key mediator of the Warburg effect, with phosphorylation at the Tyr105 site leading to a reduction in pyruvate kinase activity and promotion of the Warburg effect. Recent research has found that Atg7, a key molecule involved in autophagy, inhibits the phosphorylation of PKM2 at the Tyr105 site by blocking the binding of PKM2 to its upstream kinase FGFR1, thereby inhibiting the Warburg effect [55]. Furthermore, several CMA substrates, including PKM2, Hexokinase 2 (HK2), glucose-6 phosphate dehydrogenase (G6PD), and glyceraldehyde-3-phosphate dehydrogenase (GAPDH), have been identified. The degradation of these enzymes through CMA could potentially contribute to the inhibition of glycolysis and metabolic activity [56]. Two recent studies reported a synergistic effect when combining FLT3 inhibitors (AC220) and autophagy inhibitors (C43) [57] or (TAK165) [58] for effectively killing AML cells. It was demonstrated that under normal nutritional conditions, the simultaneous inhibition of FLT3 and autophagy can cause excessive activation of CMA. This activation, in turn, results in the degradation of the mutated form of tumor protein P53 (TP53), glyceraldehyde-3-phosphate dehydrogenase (GAPDH), an inhibitor of nuclear factor kappa B alpha (IκB-α), and HK2 protein, leading to metabolic catastrophe and cell death in AML cells [57, 58].

**Fig. 1:**
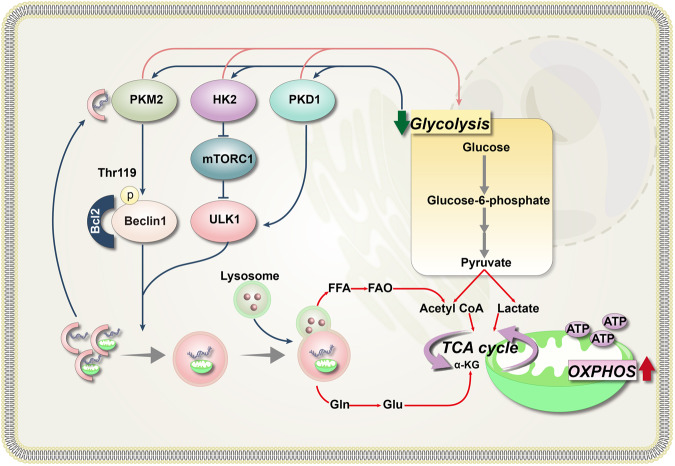
Interplay between autophagy and glycolysis in AML cells. In AML cells, the autophagy process has been found to inhibit glycolysis, potentially because autophagy contributes to the degradation of glycolysis-related enzymes [53, 57, 58]. These enzymes, including PKM2 and PDK1, not only regulate glycolysis but also induce autophagy [47, 60]. When glycolysis is suppressed, these enzymes have the ability to stimulate autophagy via an array of mechanisms [60–63]. For example, HK-II has been found to bind to and inhibit mTORC1 [63], while PKM2 is capable of phosphorylating Beclin-1 at Thr119. This phosphorylation leads to the dissociation of Beclin-1 and Bcl-2, consequently activating Beclin-1, which then triggers autophagy [60]. Autophagy can recycle excess or damaged cell organelles and degrade intracellular lipids. The resultant degradation products, including FFAs and amino acids such as Gln, can provide an essential energy source to tumor cells under starvation conditions [59]. Gln Glutamine, Glu glutamate.

Tumor cells, in contrast to normal cells, rely more on glycolysis and are thus more sensitive to low glucose levels. When faced with glucose scarcity, these cells can trigger autophagy, a survival mechanism used to counter this stress [59]. Numerous studies have underscored the role of glycolysis-related enzymes in autophagy regulation. As reported by Wang et al., PKM2 is abundantly expressed in NPM1-mutated AML, with high PKM2 levels partially upregulated by PTBP1. Notably, PKM2 has been found to stimulate autophagy via the Beclin-1 pathway, thereby promoting the survival of AML cells [60]. Similarly, pyruvate dehydrogenase kinase-1 (PDK1), another crucial glycolytic enzyme, has been demonstrated to initiate autophagy in AML cells by interacting with the key autophagy molecule ULK1 (Unc-51 like kinase-1) [47]. Moreover, other glycolysis-related enzymes, including HK2, GAPDH, and PFKFB3, have been identified as autophagy regulators. These enzymes can instigate autophagy through various mechanisms, such as the activation of AMP-activated Protein Kinase (AMPK) or the inactivation of mTOR, allowing cells to recycle cellular components and thus overcome energy supply deficiencies when glycolysis is inhibited [60–63] (Fig. 1). However, it is crucial to note that further research is required to precisely determine the roles of these enzymes in autophagy regulation within AML.

## Interplay between autophagy and amino acids metabolism in AML cells

Compared to their healthy counterparts, tumor cells not only consume glucose at an accelerated rate, but they also exhibit significantly increased demand for amino acids to support rapid growth and proliferation [64, 65]. Recent studies have reported that Leukemic stem cells (LSCs), including those in AML, heavily rely on amino acid metabolism, crucial for maintaining their stemness. Depletion of amino acids leads to impaired LSC viability. In contrast, normal hematopoietic counterparts can compensate for reduced OXPHOS through increased glycolysis when amino acids are depleted, leaving them unaffected [66–69]. This suggests that targeting amino acid metabolism could be a potential therapeutic approach for eradicating LSCs without harming normal hematopoietic counterparts.

There is a compelling link between the metabolism of amino acids such as glutamine, leucine, and proline, and the proliferation, as well as survival, of AML cells [70, 71]. Glutamine, as the most abundant amino acid in human blood, plays a crucial role in maintaining the normal functionality of various cells [72]. Numerous studies emphasize the significant dependency of AML cells on glutamine metabolism. These malignant hematopoietic cells primarily use glutamine as an alternative to the “Warburg effect” and as an ATP source for energy production [67, 73]. Cells tend to obtain glutamine via several mechanisms, such as uptake by the glutamine transporter SLC1A5 (ASCT2), internal synthesis, or the lysosomal degradation of proteins acquired via autophagy, endocytosis, and macropinocytosis [72]. In mitochondria, glutamine undergoes conversion to glutamate through the action of glutaminase (GLS) in a process known as glutaminolysis. Glutamate can be directly converted to alpha-ketoglutarate (α-KG) by glutamate dehydrogenase (GLDH) and participate in the tricarboxylic acid (TCA) cycle to support cancer cell growth and proliferation. Alternatively, glutamate can be deaminated in various reactions to provide a nitrogen source for the synthesis of non-essential amino acids, purine, and pyrimidine nucleotides [72, 74]. Moreover, glutaminolysis also contributes to the production of antioxidative molecules like glutathione and NADPH [72]. Under glutamine deprivation, cell autophagy becomes activated, which can restore cellular glutamine levels to some extent by recycling intracellular proteins and extracellular components *via* macropinocytosis [75].

**Fig. 2:**
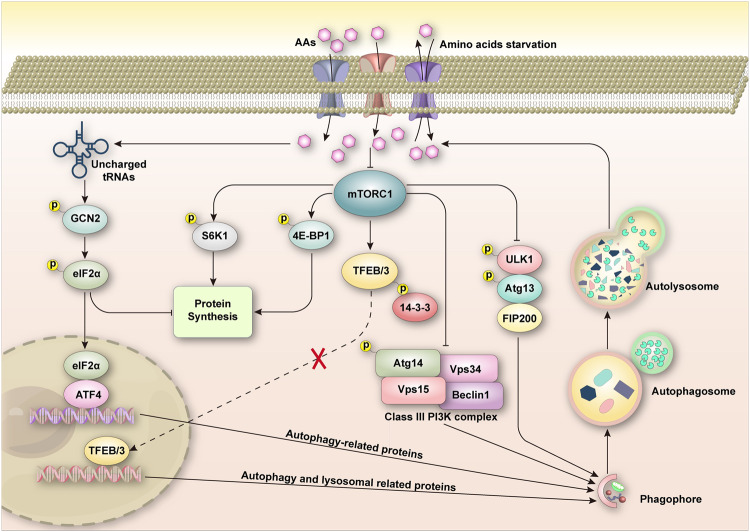
Interplay between autophagy and amino acid metabolism in AML cells. The regulation of amino acid homeostasis is largely maintained by two pathways, known as GCN2/eIF2α/ATF4 and mTORC1. These pathways orchestrate protein synthesis and autophagy in response to the cellular levels of amino acids [76]. When amino acids are in short supply, the GCN2/eIF2α/ATF4 pathway springs into action, putting a halt to protein synthesis and instigating the transcription of autophagy-related genes. This initiates the autophagy process, replenishing the amino acid pool in the cytoplasm [79]. In contrast, when amino acids are abundant, mTORC1 acts by phosphorylating downstream targets like 4E-BP1 and S6K1 to stimulate protein synthesis [83]. mTORC1 also puts a brake on the inception of autophagy by phosphorylating ULK1 and ATG13 and inhibiting the class III PI3K complex. Moreover, mTORC1 phosphorylates TFEB/3, which enhances its interaction with 14-3-3 in the cytoplasm, preventing their migration to the nucleus. However, when amino acids are lacking, mTORC1 activity is subdued, resulting in a decrease in protein synthesis and an increase in autophagy. This in turn allows TFEB/3 to relocate to the nucleus where it augments the expression of numerous lysosomal and autophagy-related genes [85, 86].

In the regulation of amino acid homeostasis, the general control non-derepressible 2 (GCN2)-activating transcription factor 4 (ATF4) and mammalian target of rapamycin complex 1 (mTORC1) play key roles. These two highly conserved pathways are responsible for modulating protein synthesis by sensing cellular amino acid levels [76] (Fig. 2). When amino acids are scarce, the GCN2/eIF2α/ATF4 pathway activates, increasing the expression of amino acid transporters on the plasma membrane [77]. This pathway also stimulates the transcription of enzymes involved in amino acid synthesis, thereby increasing the production of certain amino acids [78]. Moreover, amino acid deficiency can also initiate the transcription of autophagy genes such as Atg12, Atg5, Atg7, and Beclin-1 through the activation of the GCN2/eIF2α/ATF4 pathway, triggering the autophagy process and increasing the amino acid content in the cytoplasm [79]. In FLT3-ITD-positive AML, ATF4 activation has been discovered to be intimately linked with the proliferation of autophagy-dependent AML cells. Targeting either autophagy or ATF4 notably reduces the AML tumor load in a leukemic mouse model [9]. Currently, no drugs are available that inhibit GCN2. However, a recently reported inhibitor, known as GCN2iA, has been found to significantly improve the sensitivity of AML cells to l-asparaginase (L-ase). When used in conjunction, these two agents can synergistically trigger cell apoptosis by activating the stress-activated MAPK pathway [80].

As an important regulator of cell growth and metabolism, mTORC1 plays a crucial role in responding to changes in nutrient availability. In most primary AML samples, mTORC1 is activated through a mechanism that remains unknown [81, 82]. mTORC1 can phosphorylate downstream targets such as 4E-BP1 and S6K1, thereby positively regulating protein synthesis and cell growth [83]. Inhibition of S6K1 activity has been proven to reduce the proliferation of AML cells both in vitro and in vivo [84]. mTORC1 also plays a key role in regulating the autophagy signaling pathway and inhibits the kinase ULK1, which is involved in autophagy initiation [85]. It is well-established that mTORC1 signaling is highly sensitive to amino acids. Under nutrient-rich conditions, mTORC1 is activated. It then phosphorylates ULK1 and ATG13, which leads to the inhibition of autophagy initiation. Conversely, under nutrient-deprived conditions, mTORC1 activity is inhibited, leading to autophagy induction [85, 86]. The translocation of mTORC1 to the lysosomal membrane is the first step in its activation. This process responds to extracellular amino acid levels and is regulated by Rag GTPases. Intracellular leucine is required for the activation of Rag GTPases. Intracellular glutamine, in synergy with the transporters SLC1A5 and SLC7A5/3A2, can assist leucine entry into cells, thereby promoting mTORC1 activation and inhibiting autophagy [81]. Glutamine can also promote mTOR activation through a Rag-independent mechanism. Moreover, glutamine, glutamate, and leucine contribute to Raptor acetylation and mTOR activation by fueling the TCA cycle for the production of acetyl-CoA [59]. Depletion of glutamine, knockout of the SLC1A5 transporter, and the use of L-ase to deplete glutamine can all inhibit mTORC1 activity, thereby suppressing protein synthesis and inducing apoptosis in AML cells. However, studies have also found that while mTORC1 activity is inhibited, its inhibitory effect on ULK1 kinase is relieved, leading to autophagy initiation, which partially protects AML cells from apoptosis [81, 87]. Additionally, at appropriate levels, ammonia produced by glutaminolysis contributes to maintaining a steady-state level of autophagy in tumor cells and protects these cells from metabolic stress-induced death [88]. These studies suggest that glutamine metabolism can regulate autophagy to meet the specific needs of tumors, providing nutrients and reducing cellular stress to maintain cell survival and growth.

Over the past two decades, strategies targeting glutamine as new treatment approaches have been widely explored in AML treatment. Depletion of systemic glutamine and application of glutaminase inhibitors, glutamine antagonists/analogs, and glutamine uptake inhibitors have shown robust antileukemic responses in AML [89–95] (Table 3). However, glutamine metabolism involves multiple pathways within cells and is regulated by various factors. Moreover, leukemia cells possess high metabolic plasticity, limiting the efficacy of targeting a single amino acid pathway.

**Table 3:** Therapeutic strategies targeting glutamine metabolism in AML.

Strategy	Drug target	Drug used	Mechanism of action	Ref.
Glutamine depletion	Glutamine	l-asparaginase Erwinaze	Catalyzes the hydrolysis of asparagine and glutamine, depleting these amino acids and inhibiting cancer cell survival and proliferation	[81, 89]
Glutaminase inhibitors	GLS1	CB-839 BPTES Compound 968	Inhibiting GLS function, reducing Gln utilization and its flux into the TCA cycle	[90–92]
Glutamine antagonists/ analogs	Glutamine	DON	Irreversibly competing with Gln for enzymes’ binding site	[93]
Glutamine uptake inhibitors	SLC1A5	GPNA V-9302 (GPNA derivative)	Inhibiting glutamine transport by binding to SLC1A5	[94, 95]

*BPTES* Bis-2-(5-phenylacetamido-1,2,4-thiadiazol-2-yl)ethyl sulfide, *DON* 6-diazo-5-oxo-l-norleucine, *Gln* glutamine, *GLS* glutaminase, *GPNA* L-c-glutamyl-p-nitroanilide, *TCA* tricarboxylic acid.

## Interplay between autophagy and fatty acid metabolism in AML cells

Fatty acids are vital biomolecules for all organisms. During oxidation, fatty acids produce the highest amount of energy among all common energy substrates, either in the form of ATP or as heat. Enhanced fatty acid metabolism is a common metabolic alteration in tumor cells. Regardless of the extracellular fatty acid levels, most tumor cells can actively metabolize fatty acids to maintain energy supply. Additionally, tumor cells utilize fatty acids for the synthesis of biomembranes and crucial signaling molecules to support their own survival, invasion, metastasis, and drug resistance [96].

Recently, several studies have reported a strong association between enhanced fatty acid metabolism and malignant phenotypes in AML cells [97–99]. According to the study of Farge et al., cytarabine (AraC)-resistant acute myeloid leukemia (AML) cells are not primarily enriched in immature, quiescent cells or LSCs as previously thought. Instead, these cells demonstrate increased fatty acid oxidation (FAO), elevated CD36 expression, and a high OXPHOS gene signature [100]. In a follow-up study, Jones et al. discovered that unlike de novo AML LSCs which rely on amino acid metabolism, relapsed LSCs display a robust resistance to amino acid depletion. Remarkably, these specific cells counteract the deficiency in amino acid metabolism induced by the combined administration of venetoclax (VEN) and azacitidine (AZA), by augmenting fatty acid metabolism to fuel OXPHOS [67]. Further research has shown that inhibiting genes involved in fatty acid metabolism, such as CD36, CPT1A, and CPT1C, can significantly impede the survival and colony formation of AML cells [101]. Moreover, several studies have identified the abnormal expression of various enzymes involved in fatty acid metabolism in AML. These include ATP-citrate lyase (ACLY) [102], fatty acid synthetase (FASN) [103], and carnitine palmitoyltransferase 1 (CPT1) [104, 105], among others. Recent studies have suggested that targeting these enzymes in AML could disrupt the energy source required for AML cell growth and help overcome drug resistance, particularly in cases of relapsed and refractory AML [106–111] (Table 4).

**Table 4:** Targeting key fatty acid metabolism enzymes in AML.

Enzyme	Enzyme description	Inhibitor	Anti-AML effect in pre-clinic research	Ref.
ACC	A key enzyme that catalyzes the conversion of acetyl-CoA to malonyl-CoA	TOFA	Impairing primary human AML cells viability, synergistically inducing IDH1-mutant primary cell death with ivosidenib	[106]
ACLY	A cytosolic enzyme that catalyzes the generation of acetyl-CoA from citrate	SB-204990	Significantly reducing the proliferation of MOLM-13 and THP-1 cell lines	[102]
CPT1	A key enzyme that catalyzes the rate-limiting step of FAO	Etomoxir	Exhibiting synergistic inhibitory effects with Ara-C on AML	[100]
		Avocatin B	Synergistically enhancing ROS production and inducing apoptosis in AML cells with Ara-C	[107]
		ST1326	Exhibiting synergistic inhibitory effects with Bcl-2 inhibitor ABT199 on AML	[108]
FASN	The only human lipogenic enzyme available for de novo fatty acid synthesis	Orlistat	Exhibiting synergistic effect in promoting apoptosis in AML cells with CFZ	[109]
		TVB-3166	Exhibiting synergistic effect in promoting apoptosis in AML cells with CFZ	[109]
HMGCR	A key enzyme in the cholesterol biosynthesis pathway	Atorvastatin	Inhibiting endocytosis/pinocytosis in statin-sensitive primary human AML specimens	[110]
		Simvastatin	Blocking the chemotherapy-induced enhancement of HMGCR carrying sEVs secretion in AML cells	[111]
VLCAD	An enzyme involved in the breakdown of long-chain fatty acids	AYNE	Suppressing FAO and mitochondrial respiration, resulting in selective AML cell death	[98]

*ACC* acetyl-CoA carboxylase, *ACLY* ATP-citrate lyase, *Ara-C* cytarabine, *AYNE* avocadyne (16-heptadecyne-1,2,4-triol), *CFZ* carfilzomib, *CPT1* carnitine palmitoyltransferase 1, *DGAT* diacylglycerol acyltransferase, *FASN* fatty acid synthase, *HMGCR* hydroxymethylglutaryl-CoA reductase, *sEVs* small extracellular vesicles, *TOFA* 5-tetradecyloxy-2-furoic acid, *VLCAD* very long-chain Acyl-CoA dehydrogenase.

**Fig. 3:**
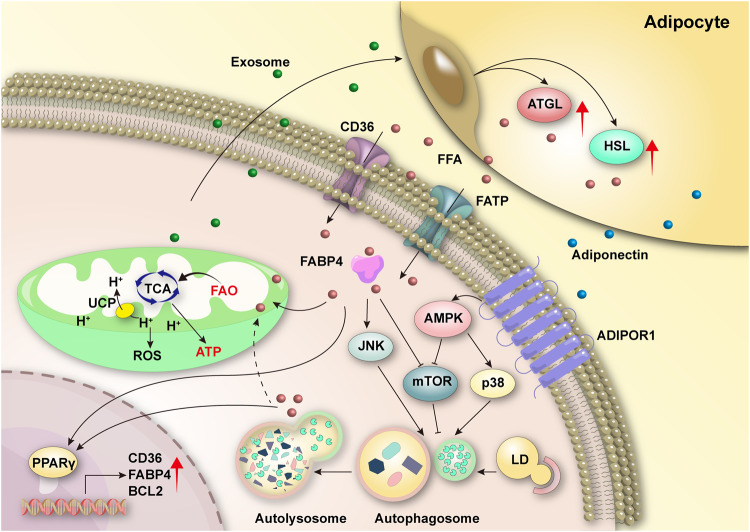
Interplay between autophagy and fatty acid metabolism in AML cells. Exosomes secreted by AML cells can enhance the expression of lipolysis genes ATGL and HSL in adipocytes, promoting lipolysis and the release of FFAs [113–115]. These FFAs are then transported into AML cells via transport proteins such as FABP4, CD36, and FATP [112, 116]. Within the mitochondria of AML cells, fatty acids are metabolized through FAO to generate energy. This process is accompanied by mitochondrial uncoupling, which leads to a reduction in ROS production, thus helping to stave off oxidative stress damage [117]. Additionally, fatty acids can activate cellular transcriptional networks involving PPARγ and its downstream target genes, including FABP4, CD36, and Bcl-2, further promoting FAO and enhancing the survival of AML cells [117]. The activation of the AMPK pathway, triggered by the binding of adipocyte-produced adiponectin to its receptor ADIPOR1, leads to autophagy. FFAs also stimulate autophagy either by activating the JNK pathway or inhibiting the mTOR pathway [117–119]. This autophagy process can breakdown LDs, and the resulting FFAs can serve as an additional energy source for leukemia cells. UCP, uncoupling protein.

In addition to de novo synthesis, cells can obtain fatty acids through exogenous lipid uptake. Adipocytes, integral components of the bone marrow stromal cells, provide a significant source of fatty acids for AML LSCs and blasts [112–116]. Kumar et al.’s studies illustrated how AML LSCs create a unique pro-inflammatory environment, either independently or *via* their secreted exosomes, promoting the differentiation of bone marrow mesenchymal stem cells (MSCs) into adipocytes. Moreover, LSC-derived exosomes enhance the expression of adipolysis genes ATGL and HSL in adipocytes, promoting lipolysis and releasing free fatty acids (FFAs). This lipolysis and release of FFAs drive FAO-dependent OXPHOS in LSCs [113–115]. Fatty acids from adipocytes are transferred to AML cells via transporter proteins like fatty acid binding protein-4 (FABP4), CD36, and others [112, 116]. Research also suggests that adipocyte-produced fatty acids can activate a transcriptional network in AML cells, involving peroxisome proliferator-associated receptor γ2 (PPARγ) and its downstream target genes, including FABP4 and CD36, thus promoting FAO and facilitating AML cell survival. Additionally, BM adipocyte-produced adiponectin activates the AMPK pathway, initiating autophagy [117]. Furthermore, free fatty acids like palmitic acid and oleic acid can promote autophagy by inhibiting mTORC1 or through the protein kinase R (PKR)-c-Jun N-terminal kinase (JNK) pathway [118, 119] (Fig. 3). Reports indicate that autophagy can degrade lipids and affect tumor lipid homeostasis [120]. However, the interaction between cellular autophagy and lipids is less well characterized than that between autophagy and amino acids or glucose.

Recent extensive research indicates that lipid droplets (LDs) within biological organisms activate a selective autophagy process known as lipophagy. During this process, LDs are encapsulated by autophagosomes. The triglycerides stored within the LDs are then degraded into FFAs by lysosomal lipases. These FFAs are metabolized through FAO, providing fuel for OXPHOS [121, 122]. FFAs can increase autophagy levels by inhibiting mTORC1 or through the PKR-JNK pathway. On the other hand, high lipid concentrations can inhibit autophagy by blocking the fusion process between autophagosomes and lysosomes or impairing lysosomal acidification and hydrolase activity [121]. In AML cells, autophagy inhibition using 3-MA or silencing key autophagy proteins Beclin-1 and ATG12 leads to a reduction in FAO. This results in reduced OXPHOS and an accumulation of LDs in AML cells, thereby inhibiting the proliferation of AML cells [120]. Further investigation showed that the disruption of mitochondria-endoplasmic reticulum (ER) contact sites (MERCs) mimics OXPHOS inhibition. This suggests that mitochondria can control autophagy through the regulation of MERCs, finely tuning lipid degradation to fuel OXPHOS, which supports proliferation in leukemia [120]. Given the unique capacity of tumor cells to leverage such regulatory mechanisms, understanding the role of MERCs in AML could provide valuable insights into the disease’s metabolic plasticity and resilience. It also raises the intriguing possibility that targeting these mitochondrial processes could lead to novel therapeutic strategies in AML by disrupting the tumor’s metabolic balance or enhancing the efficacy of existing treatments through combinatorial approaches. Future research could focus on elucidating the precise molecular mechanisms by which MERCs control autophagy and OXPHOS in AML, potentially unveiling new targets for intervention.

Studies have shown that there is a close relationship between classical lipolysis and lipophagy. The ATGL plays a crucial role in this interaction [123]. ATGL is a key enzyme that regulates the breakdown of triglycerides (TAGs). It converts them into FFAs. ATGL enhances the cell’s ability to mobilize and metabolize fatty acids through the SIRT1/PPAR-γ coactivator 1-α (PGC-1α)/peroxisome proliferator-activated receptor-α (PPAR-α) pathway [124]. Additionally, ATGL facilitates the degradation of LDs by interacting with the autophagy marker protein LC3. It initiates the process of lipophagy. These findings highlight the importance of ATGL in coordinating both lipolysis and lipophagy [125, 126].

In contrast, FASN inhibits autophagy. FASN is a key rate-limiting enzyme in fatty acid synthesis. It catalyzes the consecutive condensation reaction of acetyl-CoA and malonyl-CoA to generate fatty acids. Studies have reported that the expression of FASN in AML cells is significantly higher than in normal hematopoietic cells. FASN can inhibit cell autophagy by activating the mTOR pathway. This inhibits the normal differentiation of cells [103]. Treatment with all-trans retinoic acid (ATRA) can promote autophagy. It degrades FASN through autophagy, further enhancing autophagic activity. This accelerates the differentiation of acute promyelocytic leukemia (APL) cells through this positive feedback mechanism [103]. It is clear that autophagy plays different and even opposing roles in the development and treatment of AML. Further in-depth and extensive research is needed. This will help determine how to harness the beneficial effects of autophagy and avoid its detrimental effects in AML treatment.

## Autophagy is involved in the metabolism coupling between TME and AML

Recent studies have demonstrated that cancer cells can remodel the microenvironment into a self-reinforcing tumor microenvironment (TME) to support their survival and proliferation [127, 128]. The TME comprises various stromal cells, including mesenchymal cells, adipocytes, endothelial cells, neuroendocrine cells, immune-inflammatory cells, and notably, cancer-associated fibroblasts (CAFs). CAFs play a significant role in cancer progression due to their abundance and interaction with cancer cells [129]. Under oxidative stress, CAFs and other stromal cells exhibit a conspicuous catabolic phenotype. Metabolites such as ketones, pyruvate, lactate, amino acids, and nucleotides are produced *via* glycolysis and autophagy in these stromal cells. These metabolites are then released into the TME and taken up by cancer cells to fuel their oxidative metabolism and support their rapid proliferation. This phenomenon is commonly referred to as the “reverse Warburg” effect [130].

Recent studies have revealed the presence of the “reverse Warburg” effect across various forms of leukemia, including AML [131]. In a comparative analysis carried out by Henkenius et al., the metabolic reactions of AML and SCLC cells when exposed to cytarabine or sorafenib were scrutinized. The findings shed light on the different strategies these two cell types employ to evade drug toxicity. SCLC cells shift completely to glycolysis when subjected to chemotherapy, leveraging the Warburg effect to counter drug toxicity. In stark contrast, AML cells keep up oxidative metabolism following drug exposure, implying their evasion of drug toxicity *via* the “reverse Warburg” effect. This further highlights the pivotal role that oxidative pathways play in the bioenergetics of AML cells [131]. Subsequent studies demonstrated that the inhibition of the mitochondrial oxidative metabolism boosts apoptosis in FLT3-ITD^+^ AML. However, AML cells can swiftly adjust by drawing lactate from the extracellular microenvironment. By blocking Monocarboxylic Acid Transporter 1 (MCT1), lactate transport inhibition significantly heightens the sensitivity of AML cells to mitochondrial electron transport chain (ETC) complex II inhibition [132].

Mounting evidence suggests that oxidative stress may play a pivotal role in the metabolic reprogramming of the “reverse Warburg” effect. ROS derived from cancer cells are released into the TME, inducing oxidative stress in CAFs and other stromal cells [133]. Hypoxia Inducible Factor 1α (HIF-1α), a crucial transcriptional regulator of hypoxic responses, is upregulated under oxidative stress, which subsequently triggers a hypoxic response, enhances the transcription of angiogenic factors, such as vascular endothelial growth factor (VEGF), and mediates autophagy, mitophagy, and aerobic glycolysis [134]. Given these findings, some researchers have proposed the term “Autophagic Tumor Stroma Model of Cancer Metabolism,” which extends the concept of the “reverse Warburg” effect to autophagy/mitophagy in the tumor stroma [133]. Currently, research on the role of stromal cell autophagy in the metabolic reprogramming of AML cells is incredibly scarce. However, a recent study by Piya et al. illustrated that co-culturing AML cells with stromal cells increases autophagy and chemoresistance in AML cells exposed to cytarabine and idarubicin. This effect is reversed upon Atg7 knockout, and further amplified by concurrent Atg7 knockdown in both AML and stromal cells. These findings strongly suggest that Atg7, as well as microenvironmental autophagy, may play a significant role in AML chemoresistance [135]. Although the exact role and mechanism of autophagy in the metabolic coupling between TME and AML still require comprehensive and in-depth research, the existing evidence suggests that targeting autophagy to disrupt the metabolic coupling between TME and AML may represent a promising strategy for circumventing the protective effects of stromal cells in AML.

## Conclusions

In the pathological mechanism of AML, the interaction and coordination between autophagy and metabolism play a vital role in maintaining intracellular physiological homeostasis, which is potentially a significant factor behind the resistance exhibited by AML cells during chemotherapy. Consequently, the dual regulation of energy metabolism and autophagy within AML cells has emerged as a promising new therapeutic strategy in recent years. However, it’s crucial to note that AML is a highly heterogeneous disease, where autophagy may assume different, or even diametrically opposite roles in various types and stages of AML cells. Furthermore, the energy metabolism of AML cells demonstrates high heterogeneity, complexity, and diversity, suggesting that the regulatory role of autophagy within it is also complex and variable. Given AML’s complexity, the advancement of personalized treatment becomes particularly necessary. This necessitates the application of a series of technical methodologies, such as gene sequencing to determine the disease’s specific genotype, cell biology experiments to observe the state of autophagy and metabolism within cells, and drug sensitivity tests to determine the most effective drug combinations. These steps will enable us to discern the role of autophagy in specific AML cases and formulate the most appropriate treatment strategy based on that information.

In AML, the key to autophagy-targeted treatment lies in striking a delicate balance and meticulously adjusting intervention measures according to the metabolic profile and specific stage of individual AML patients, thus accurately determining when to inhibit and when to activate autophagy. By integrating autophagy regulation with drug intervention, we can disrupt the favorable conditions autophagy provides for malignant cells, and circumvent its metabolic reprogramming adaptation strategy, thereby enhancing the sensitivity of AML cells to existing treatments. Moreover, to achieve personalized treatment, we need to delve deeper into the interactions between leukemia-specific autophagy and metabolism, and develop biomarkers based on autophagy for precise patient stratification. To skillfully navigate this complex interaction network, future research should broaden its focus to include interactions between autophagy inhibitors and activators, metabolic pathway modulators, and systemic and cellular metabolic signals. By integrating metabolic reprogramming and autophagy-targeted treatment into the established paradigm of AML treatment, we can expect a transformative impact on AML clinical management, offering more hope and potential for the future of AML patients.

## Data Availability

Data sharing is not applicable to this article as no datasets were generated or analyzed during this study.
